# Contribution of Genome-Wide Association Studies to Scientific Research: A Bibliometric Survey of the Citation Impacts of GWAS and Candidate Gene Studies Published during the Same Period and in the Same Journals

**DOI:** 10.1371/journal.pone.0051408

**Published:** 2012-12-11

**Authors:** Yohann Mansiaux, Fabrice Carrat

**Affiliations:** 1 Epidemiology, Information System, Modeling, UMR-S 707, University Paris 6-UPMC, Paris, France; 2 Inserm U707, Paris, France; 3 Public Health Unit, Saint-Antoine Hospital, Paris, France; The Children’s Hospital of Philadelphia, United States of America

## Abstract

In genetic epidemiology, genome-wide association studies (GWAS) are used to rapidly scan a large set of genetic variants and thus to identify associations with a particular trait or disease. The GWAS philosophy is different to that of conventional candidate-gene-based approaches, which directly test the effects of genetic variants of potentially contributory genes in an association study. One controversial question is whether GWAS provide relevant scientific outcomes by comparison with candidate-gene studies. We thus performed a bibliometric study using two citation metrics to assess whether the GWAS have contributed a capital gain in knowledge discovery by comparison with candidate-gene approaches. We selected GWAS published between 2005 and 2009 and matched them with candidate-gene studies on the same topic and published in the same period of time. We observed that the GWAS papers have received, on average, 30±55 citations more than the candidate gene papers, 1 year after their publication date, and 39±58 citations more 2 years after their publication date. The GWAS papers were, on average, 2.8±2.4 and 2.9±2.4 times more cited than expected, 1 and 2 years after their publication date; whereas the candidate gene papers were 1.5±1.2 and 1.5±1.4 times more cited than expected. While the evaluation of the contribution to scientific research through citation metrics may be challenged, it cannot be denied that GWAS are great hypothesis generators, and are a powerful complement to candidate gene studies.

## Introduction

The first genome-wide association study (GWAS) was published in 2005 and identified a genetic variant associated with a higher risk of age-related macular degeneration [1]. The completion of the Human Genome Project [2] and the HapMap Project [3] yielded tools to identify common genetic variations, mainly single-nucleotide polymorphisms (SNPs), associated with many traits or diseases. In 2008, the National Human Genome Research Institute (NHGRI) published an online catalog of Genome-Wide Association Studies [4], listing genetic marker-trait associations published in GWAS. As of 01 November 2012, this database included 1416 publications and 7688 disease- or trait-associated genetic variants.

These “wide association” approaches, initially devoted to genetic associations, have since been extended to transcriptomic [5], [6], metabolomic [7] and environmental factors (chemical toxicants, pollutants or nutrients) [8]. While examination of many common genetics variants [9] or environmental exposures [10] is relevant because of their known role in the development of diseases, one may wonder if simultaneous analysis of such an overwhelming amount of data using data-driven methods leads to a significant value in the generation of knowledge, compared with the classical hypothesis-driven candidate-gene studies.

To answer this question, we conducted a bibliometric survey using two citation metrics, the citation count and the crown index, as a surrogate marker for the contribution to the scientific research of GWAS and candidate-gene studies focusing on the same trait or disease and published during the same time period in a predefined set of journals.

## Materials and Methods

### Data

We searched for GWAS published between 2005 and 2009 in *Nature, Science* and journals ranked among the 10 leading journals according to the 2010 Journal Impact Factors (JIFs), in the “Genetics & Heredity” or “Medicine, General & Internal” categories (see Table S1). Meta-analyses, reviews and other publication types were excluded. GWAS had to be performed in humans.

The first search was conducted in the MEDLINE database with the search terms “GWAS”, “Genome-Wide Association Study”, “Genome-Wide Association Studies”, “Genome Wide Association Study”, “Genome Wide Association Studies”, “Genomewide Association Study” and “Genomewide Association Studies”, “Whole Genome Association Study”, “Whole Genome Association Studies”, “WGAS”, “WGA study” and “WGA studies”. To ensure an exhaustive selection process, we retrieved from the NHGRI Catalog of Genome-Wide Association Studies those studies not identified by the MEDLINE search.

The search for candidate-gene studies was conducted using BioPython [11], a set of freely available tools for biological computation, written in Python. For each paper in the GWAS group, we searched for papers focusing on the same disease or trait and published the same year in the same set of journals. We selected the paper that allowed us to reduce both the difference between the 2010 JIFs of the journals in which the GWAS and candidate-gene study were published, and to reduce the interval (in months) between the 2 publication dates. The studied trait or disease was, if available, retrieved from the NHGRI Catalog of Genome-Wide Association Studies, or was otherwise added manually.

### Citation Metrics

The “quantity” of knowledge provided by the GWAS and candidate-gene studies was assessed with 2 metrics, namely the citation count and the crown index, computed 1 and 2 years after the publication dates. The citation count was recorded in the Web of Science database. The crown index depends on the expected citation rate, which indicates how frequently cited a paper is expected to be according to its year of publication, its publication type, and the journal in which it was published. The crown index is the ratio of the citation count to the expected citation rate.

Our main objective was to explore the differences of citation metrics between the GWAS and candidate-gene studies. To help interpretation of findings, we also compared candidate-gene studies derived from GWAS with those not derived from GWAS; we investigated the association between the reported P-value and citation metrics and we evaluated the impact of residual confounding, i.e. whether residual differences in impact factor or publication dates despite matching may have influenced the results.

### Statistical Analysis

The statistical analysis was conducted using R 2.13.0. The alpha level for all tests was 0.05.

Wilcoxon signed-rank tests for paired samples were used to test differences of quantitative variables between GWAS and candidate gene studies. Linear mixed-effects models were used to assess the effect of the approach used, “Genome-Wide” or “candidate gene”, on the crown index. A random effect was included to account for the correlation between the citation metrics in each pair of papers. The models were fitted with and without the effect of the approach and likelihood ratio tests were performed to compare the two fits.

GWAS and candidate-gene studies statistical tests for association do not usually use the same threshold to assess the statistical significance of an association. For example, the significance threshold in GWAS is often defined between 10^−8^ and 10^−6^ while it is usually close to 10^−2^ in candidate-gene studies. To investigate the association of P-values with citation metrics, we therefore calculated in each study the ratio between the reported P-value and the corresponding probability threshold used to conclude statistical significance (hereafter P-ratio). We explored the association between the log-transformed P-ratio, the type of study and the crown indexes using mixed models as described above.

We finally used linear regression models to assess the impact of the difference between the 2010 JIFs and of the interval between the publication dates on the difference in the crown indexes within each pair of papers.

Note: Summary statistics are reported as mean (± sd). The estimates of the linear mixed-effects models are reported as estimate (± se).

## Results

### GWAS and Candidate-gene Study Selection

**Figure 1:**
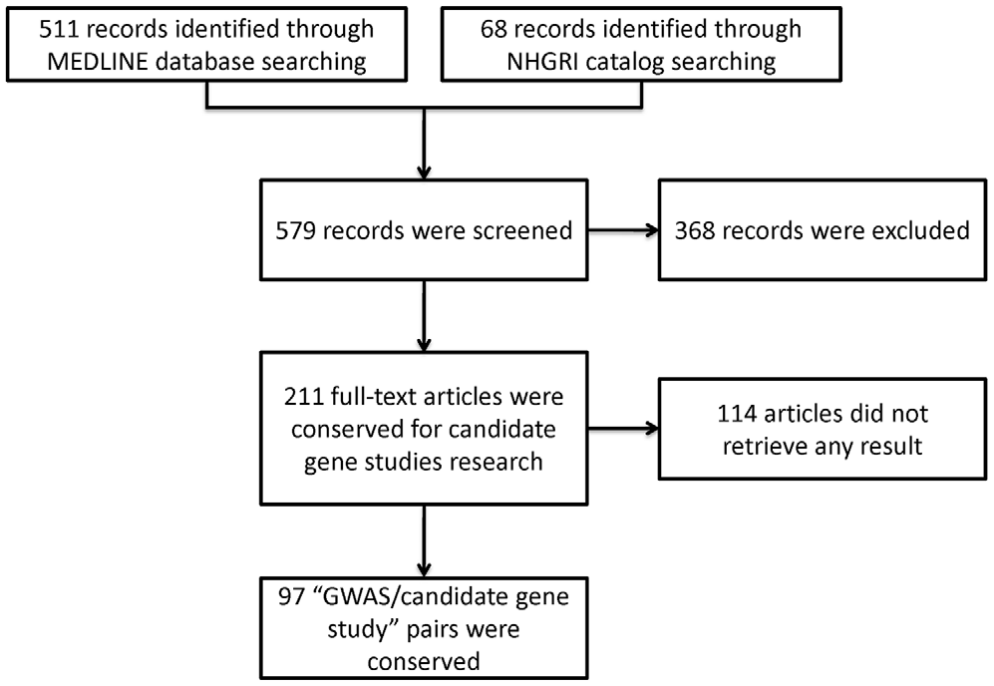
Study flow diagram.

The initial search in the MEDLINE database identified 511 articles, and 68 additional articles were identified in the NHGRI Catalog. A total of 211 GWAS were selected, of which a candidate-gene study was identified in 97 cases (62 different papers) (see Tables S2, S3 and S4 for summary statistics of the 97 GWAS/candidate-gene study pairs and Tables S5, S6, S7 and S8 for the PMID PUBMED identifiers of the articles used in the survey). The full article selection process is summarized in Figure 1. Of the 62 candidate-gene selected, 23 of them (37%) were derived from former GWAS (not necessarily from the GWAS with which the candidate-gene study was paired).

### Citation Metrics Analysis

**Figure 2:**
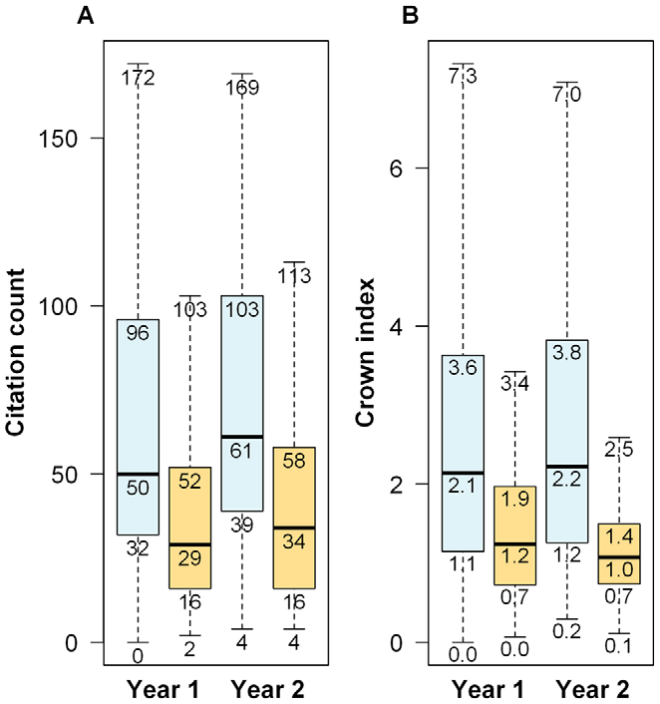
Boxplots of the citation count and of the crown index. Distributions of the citation count and of the crown index are depicted on panel A and panel B, respectively, 1 year (Year 1) and 2 years (Year 2) after the publication dates. Blue boxes refer to GWAS and orange boxes to candidate-gene studies. The boxplots depict five statistics: the sample minimum, the lower quartile, the median, the upper quartile, and the sample maximum.

We observed that the GWAS papers were more frequently cited than the candidate-gene studies, whatever the metric used (see Table 1 and Figure 2). This was confirmed by comparing the citation counts and the crown indexes, 1 and 2 years after the publication dates. The two-sided Wilcoxon signed-rank tests were all significant (P value  = 1.8×10^−8^ and 7.8×10^−11^ for the citation counts; P value  = 2.6×10^−7^ and 4.7×10^−12^ for the crown indexes). The distribution of the crown indexes were not significantly different between the candidate-gene studies derived from GWAS, compared with those not derived from GWAS, both 1 year after the publication date (Wilcoxon test P value  = 0.67) and 2 years after the publication date (Wilcoxon test P value  = 0.46).

**Table 1:** Summary statistics of the citation metrics of the “GWAS” and “candidate-gene study” papers.

Group	GWAS	Candidate-gene studies
citation count 1 year after the publication date	69.1±54.9	38.7±35.5
citation count 2 years after the publication date	85.3±69.5	45.6±47.8
crown index 1 year after the publication date	2.8±2.4	1.5±1.2
crown index 2 years after the publication date	2.9±2.4	1.5±1.4

Mean±sd.

Assessment of the effect of the “genome-wide” or “candidate-gene” approach on the crown indexes based on linear mixed-effects models confirmed that this metric increased in GWAS papers compared with candidate-gene papers. The estimates of the “genome-wide” effect on the crown indexes were 1.31 (±0.23, likelihood ratio test P value  = 3.3×10^−6^) and 1.45 (±0.19, likelihood ratio test P value  = 4.3×10^−7^), 1 and 2 years after the publication dates.

The average log transformed P-ratio was lower in the GWAS group than in the candidate-gene group : −25.8 (±54.7) for the GWAS group, −12.6 (±24.9) for the candidate-gene group (Wilcoxon test P value  = 6.9×10^−9^). The estimates of the mixed models assessing the effect of the log transformed P-ratio on the crown index 1 and 2 years after the publication date were 3.0×10^−13^ (±8.6×10^−8^, P value  = 0.97) and −4.4×10^−13^ (±1.0×10^−7^, P value  = 0.98).

There was no evidence for residual confounding: the average difference between the 2010 JIFs of the journals in which the paired papers were published was 1.5 (±15.9, Wilcoxon test P value  = 0.24) and the average interval between the publication dates was 2.7 (±2.9) months (Wilcoxon test P value  = 0.71). The effects of these differences on the difference in the crown indexes within each pair of papers in linear models were not significant, either one year after publication (P value  = 0.56 for the difference in 2010 JIFs, and P value  = 0.37 for the interval between the publication dates) or 2 years after publication (P value  = 0.90 for the difference in 2010 JIFs and P value  = 0.36 for the interval between the publication dates).

## Discussion

We showed that GWAS had a higher impact in the scientific literature than candidate gene studies. We assume that our study selection process, although not systematic, produced robust results by focusing on the genetic analyses published in the papers with the most important impact factors, and that our matching process did not alter the quality of the results provided.

The use of citations metrics to assess the ability of GWAS and candidate-gene studies to generate knowledge can be questioned. Indeed it was not possible to directly link the citation metrics to the relevance or the applicability of the scientific outcomes provided by these studies, or to their contribution to understanding the disease’s etiology. An in-depth analysis of the papers citing the GWAS and the candidate-gene studies would have been required to observe how the findings provided by those papers were used. However, we can consider that the citation metrics used are good markers for “research” generation, and therefore good, albeit indirect, markers for knowledge generation. Indeed, we observed that nearly 40% of the candidate-gene studies selected in our survey were inspired by hypotheses derived from GWAS.

The investigation of the potential association of the reported P values in the GWAS and the candidate-gene studies and of the crown index did not permit to show a significant relationship. Although the statistical threshold to assess significance is stricter in GWAS because of the multiplicity of tests, their findings were not associated with a higher scientific impact, and not associated with a higher credibility for follow-up.

### Conclusions

We have explored the impact of GWAS and candidate-gene studies through 2 citation metrics and observed that the GWAS papers were more cited than the candidate-gene papers, whatever the metrics used. If it cannot formally be claimed that the knowledge generated by GWAS outweighs that provided by classical genetic association studies, we can say that this manuscript provides evidence, at least indirect evidence, that GWAS contribute to knowledge production by allowing candidate-gene studies to focus on credible candidates.

## Supporting Information

Table S1 **2010 Journal Impact Factors of the Journals used for the MEDLINE database and NHGRI Catalog research.**
(PDF)

Table S2 **Years of publication of the 97 pairs of papers in the “GWAS” and “candidate-gene studies” groups.**
(PDF)

Table S3 **Journals of publication of the “GWAS” papers.**
(PDF)

Table S4 **Journal of publication of the “candidate-gene studies” papers.**
(PDF)

Table S5 **PMID PUBMED identifiers of the 511 articles identified through MEDLINE research.**
(XLS)

Table S6 **PMID PUBMED identifiers of the 68 articles identified through NHGRI catalog research.**
(XLS)

Table S7 **PMID PUBMED identifiers of the 211 GWAS articles conserved for candidate-gene study research.**
(XLS)

Table S8 **PMID PUBMED identifiers of the 97 GWAS/candidate-gene study pairs.**
(XLS)

## References

[1] Klein RJ, Zeiss C, Chew EY, Tsai JY, Sackler RS, et al (2005) Complement factor H polymorphism in age-related macular degeneration. Science 308: 385–389. 15761122 10.1126/science.1109557 PMC1512523

[2] Venter JC, Adams MD, Myers EW, Li PW, Mural RJ, et al (2001) The sequence of the human genome. Science 291: 1304–1351. 11181995 10.1126/science.1058040

[3] Altshuler DM, Gibbs RA, Peltonen L, Dermitzakis E, Schaffner SF, et al (2010) Integrating common and rare genetic variation in diverse human populations. Nature 467: 52–58. 20811451 10.1038/nature09298 PMC3173859

[4] Hindorff L, MacArthur JEBI, Wise A, Junkins H, Hall P, et al. (2009) A Catalog of Published Genome-Wide Association Studies. National Human Genome Research Institute.

[5] Tang B, Di Lena P, Schaffer L, Head SR, Baldi P, et al (2011) Genome-wide identification of Bcl11b gene targets reveals role in brain-derived neurotrophic factor signaling. PLoS One 6: e23691. 21912641 10.1371/journal.pone.0023691 PMC3164671

[6] Marques FZ, Campain AE, Tomaszewski M, Zukowska-Szczechowska E, Yang YH, et al (2011) Gene expression profiling reveals renin mRNA overexpression in human hypertensive kidneys and a role for microRNAs. Hypertension 58: 1093–1098. 22042811 10.1161/HYPERTENSIONAHA.111.180729

[7] Yap IK, Brown IJ, Chan Q, Wijeyesekera A, Garcia-Perez I, et al (2010) Metabolome-wide association study identifies multiple biomarkers that discriminate north and south Chinese populations at differing risks of cardiovascular disease: INTERMAP study. J Proteome Res 9: 6647–6654. 20853909 10.1021/pr100798r PMC3117148

[8] Patel CJ, Bhattacharya J, Butte AJ (2010) An Environment-Wide Association Study (EWAS) on type 2 diabetes mellitus. PLoS One 5: e10746. 20505766 10.1371/journal.pone.0010746 PMC2873978

[9] Rappaport SM (2011) Implications of the exposome for exposure science. J Expo Sci Environ Epidemiol 21: 5–9. 21081972 10.1038/jes.2010.50

[10] Rappaport SM, Smith MT (2010) Epidemiology. Environment and disease risks. Science 330: 460–461. 20966241 10.1126/science.1192603 PMC4841276

[11] Cock PJ, Antao T, Chang JT, Chapman BA, Cox CJ, et al (2009) Biopython: freely available Python tools for computational molecular biology and bioinformatics. Bioinformatics 25: 1422–1423. 19304878 10.1093/bioinformatics/btp163 PMC2682512

