# Supplementary material for: Contribution of Genome-Wide Association Studies to Scientific Research: A Bibliometric Survey of the Citation Impacts of GWAS and Candidate Gene Studies Published during the Same Period and in the Same Journals
Source: PLoS One. 2012 Dec 11;7(12):e51408. doi: 10.1371/journal.pone.0051408 (PMC3519865; doi:10.1371/journal.pone.0051408)
Supplement: Table S1 — 2010 Journal Impact Factors of the Journals used for the MEDLINE database and NHGRI Catalog research. (PDF) [file pone.0051408.s001.pdf]

Table S1 2010 Journal Impact Factors of the Journals used for the MEDLINE database and NHGRI Catalog research

**Journal**	**2010 Journal Impact Factor**
**Medicine, General & Internal**	
New Engl J Med	53.486
Lancet	33.633
Jama-J Am Med Assoc	30.011
Ann Intern Med	16.729
Plos Med	15.617
Brit Med J	13.471
Annu Rev Med	12.457
Arch Intern Med	10.639
Can Med Assoc J	9.015
Cochrane Db Syst Rev	6.186
**Genetics & Heredity**	
Nat Genet	36.377
Nat Rev Genet	32.745
Annu Rev Genet	21.774
Annu Rev Genom Hum G	17.182
Trends Ecol Evol	14.448
Genome Res	13.588
Gene Dev	12.889
Am J Hum Genet	11.680
Trends Genet	11.364
Plos Genet	9.543
**Multidisciplinary Sciences**	
Nature	36.104
Science	31.377
