# Supplementary material for: Contribution of Genome-Wide Association Studies to Scientific Research: A Bibliometric Survey of the Citation Impacts of GWAS and Candidate Gene Studies Published during the Same Period and in the Same Journals
Source: PLoS One. 2012 Dec 11;7(12):e51408. doi: 10.1371/journal.pone.0051408 (PMC3519865; doi:10.1371/journal.pone.0051408)
Supplement: Table S2 — Years of publication of the 97 pairs of papers in the “GWAS” and “candidate-gene studies” groups. (PDF) [file pone.0051408.s002.pdf]

Table S2 Years of publication of the 97 pairs of papers in the "GWAS" and "candidate-gene studies" groups

**Year of publication**	**2005**	**2006**	**2007**	**2008**	**2009**
**Number of papers**	2	2	25	23	45
