# Supplementary material for: Contribution of Genome-Wide Association Studies to Scientific Research: A Bibliometric Survey of the Citation Impacts of GWAS and Candidate Gene Studies Published during the Same Period and in the Same Journals
Source: PLoS One. 2012 Dec 11;7(12):e51408. doi: 10.1371/journal.pone.0051408 (PMC3519865; doi:10.1371/journal.pone.0051408)
Supplement: Table S3 — Journals of publication of the “GWAS” papers. (PDF) [file pone.0051408.s003.pdf]

Table S3 Journals of publication of the "GWAS" papers

| **Journal** | **Number of papers** |
|---|---|
| Am J Hum Genet | 6 |
| Gene Dev | 1 |
| Genome Res | 1 |
| Jama-J Am Med Assoc | 1 |
| Nature | 7 |
| Nat Genet | 59 |
| New Engl J Med | 5 |
| Plos Genet | 9 |
| Science | 8 |
