# Supplementary material for: Contribution of Genome-Wide Association Studies to Scientific Research: A Bibliometric Survey of the Citation Impacts of GWAS and Candidate Gene Studies Published during the Same Period and in the Same Journals
Source: PLoS One. 2012 Dec 11;7(12):e51408. doi: 10.1371/journal.pone.0051408 (PMC3519865; doi:10.1371/journal.pone.0051408)
Supplement: Table S4 — Journal of publication of the “candidate-gene studies” papers. (PDF) [file pone.0051408.s004.pdf]

Table S4 Journal of publication of the "candidate-gene studies" papers

**Journal**	**Number of papers**
Am J Hum Genet	16
Nature	3
Nat Genet	47
New Engl J Med	11
Plos Genet	11
Science	9
